# Assessment of risk factors for postoperative cognitive dysfunction after coronary artery bypass surgery: a single-center retrospective cohort study

**DOI:** 10.1042/BSR20190719

**Published:** 2021-02-22

**Authors:** Yongtao Sun, Hai Feng, Ting Zou, Ming Hou, Yanwu Jin, Changping Gu, Yuelan Wang, Juan Li, Mengjie Liu, Min Zhang, Yalei Gao

**Affiliations:** 1 Department of Anesthesiology, The First Affiliated Hospital of Shandong First Medical University & Shandong Provincial Qianfoshan Hospital, Ji’nan, Shandong 250014, China; 2 Department of Anesthesiology, The Second Hospital of Shandong University, Ji’nan, Shandong 250014, China; 3 Department of Anesthesiology, Southern District of Anhui Provincial Hospital, Hefei, Anhui 230071, China

**Keywords:** Coronary artery bypass grafting, Neuropsychological test, Postoperative cognitive dysfunction, Risk factors

## Abstract

Aim: To find out risk factors for postoperative cognitive dysfunction (POCD) after coronary artery bypass grafting (CABG), and to provide basis for clinical prevention of POCD. A total of 88 patients who underwent CABG were surveyed with Telephone Questionnaire (TICS-M) for their cognitive impairment after 3, 7, 21, 90, 180 days post-surgery. The occurrence of POCD was diagnosed by Neuropsychological Battery which included Vocabular Learning Test (VLT), Wisconsin Card Sorting Test (WCST), Trail Making Test (TMT) and Symbol Digit Modalities Test (SDMT). The preoperative, intraoperative and postoperative risk factors were assessed by the χ^2^ or *t* test. Multivariate analysis was used to study the correlation between the risk factors and the occurrence of POCD. Age, aortic plaque, carotid artery stenosis, cerebrovascular disease, anesthesia time, the rate of decline in intraoperative hemoglobin concentration (ΔHb) and systemic inflammatory response syndrome (SIRS) score on postoperative day 2 had statistically significant (*P*<0.05) influence on the occurrence of POCD. Aortic plaque, carotid artery stenosis, anesthesia time and SIRS score (odds ratio (OR) value > 1, *P*<0.05) are the risk factors for POCD. The incidence of day-21 and -180 POCD was approximately 26.1 and 22.7%, respectively.

## Introduction

Postoperative cognitive dysfunction (POCD) is a common neurological complication of coronary artery bypass grafting (CABG) that is characterized by impaired memory and learning ability, loss of attention and declined information processing speed [1–3]. Clinical diagnosis of POCD mainly relies on a series of neuropsychological tests at present. Whereas, due to the different sensitivities and specificities of the detection methods, and the burden of adding additional tests, the diagnosis criteria of POCD is not unified. Thus, a more effective and simple diagnostic criteria should be proposed for POCD. By far the pathophysiology of POCD has not been clearly elucidated, but it is widely believed that risk factors for CABG patients developing POCD include age, education, hypertension, aortic plaque, carotid artery stenosis, cerebrovascular disease, anesthesia time, length of surgery, blood oxygen saturation in brain surgery and systemic inflammation. However, the risk factor varies among different studies because of the unified diagnosis criteria of POCD, procedure of the analysis, and subjective effects [4]. Hence, systemic in-depth studies to characterize the high-risk factors and clarify the effective preventative measures and behavior evaluation method of POCD have been called for.

Previous studies suggested that at least 30% of patients may experience cognitive dysfunction after cardiac surgery, and ultimately affect the recovery and the quality of life in patients who had undergone CABG [4,5]. Initially, POCD was considered as an intra- and immediate event during post-operation, with further research in recent years, POCD was gradually found to persist for months or years after surgery. The incidence of short-term POCD after heart surgery is 65%, whereas 40% of patients still suffered from POCD 3–6 months after surgery [6,7]. However, the affection of short-term and long-term cognitive impairment in cardiovascular diseases (CVDs) patients who underwent CABG surgery are yet to be identified.

To address these issues, we performed a retrospective cohort study of CVD patients who have undergone CABG surgery. In our study, a dynamic observation was first implemented to determine the incidence of POCD in patients who received CABG surgery for 21 and 180 days, latter, the risk factors of POCD were analyzed in order to provide experimental basis for the establishment of the preventive measures and a more convenient and feasible diagnostic criteria.

## Materials and methods

After the approval of the ethics committee of Qianfoshan Hospital affiliated to Shandong University, the patients signed an informed consent form. A total of 129 patients who were aged 40–80 years and scheduled for elective CABG surgery between June 2015 and May 2017 were screened, and among them, the number of male and female patients was 76 and 53 respectively, the average age was 66.4 ± 9.3.

### Inclusion criteria

Patients with the following criteria were deemed eligible for enrollment in the present study: (1) diagnosed as Coronary Heart Diseases (CHD) according to the criteria by the World Health Organization; (2) multiple coronary stenosis (vascular stenosis > 75%), and elective CABG was manipulated; (3) tested with Mini-mental state examination (MMSE) and proved with no cognitive impairment: illiteracy > 17 points, elementary school (years of education ≤ 6 years) > 20 points, middle school or higher > 25 points [8]; (4) significant improvement in the degree of vascular stenosis after CABG surgery was observed: angina and cardiac dysfunction can be effectively relieved and (5) voluntary participation in the test and signed the informed consent.

### Exclusion criteria

**Figure 1:**
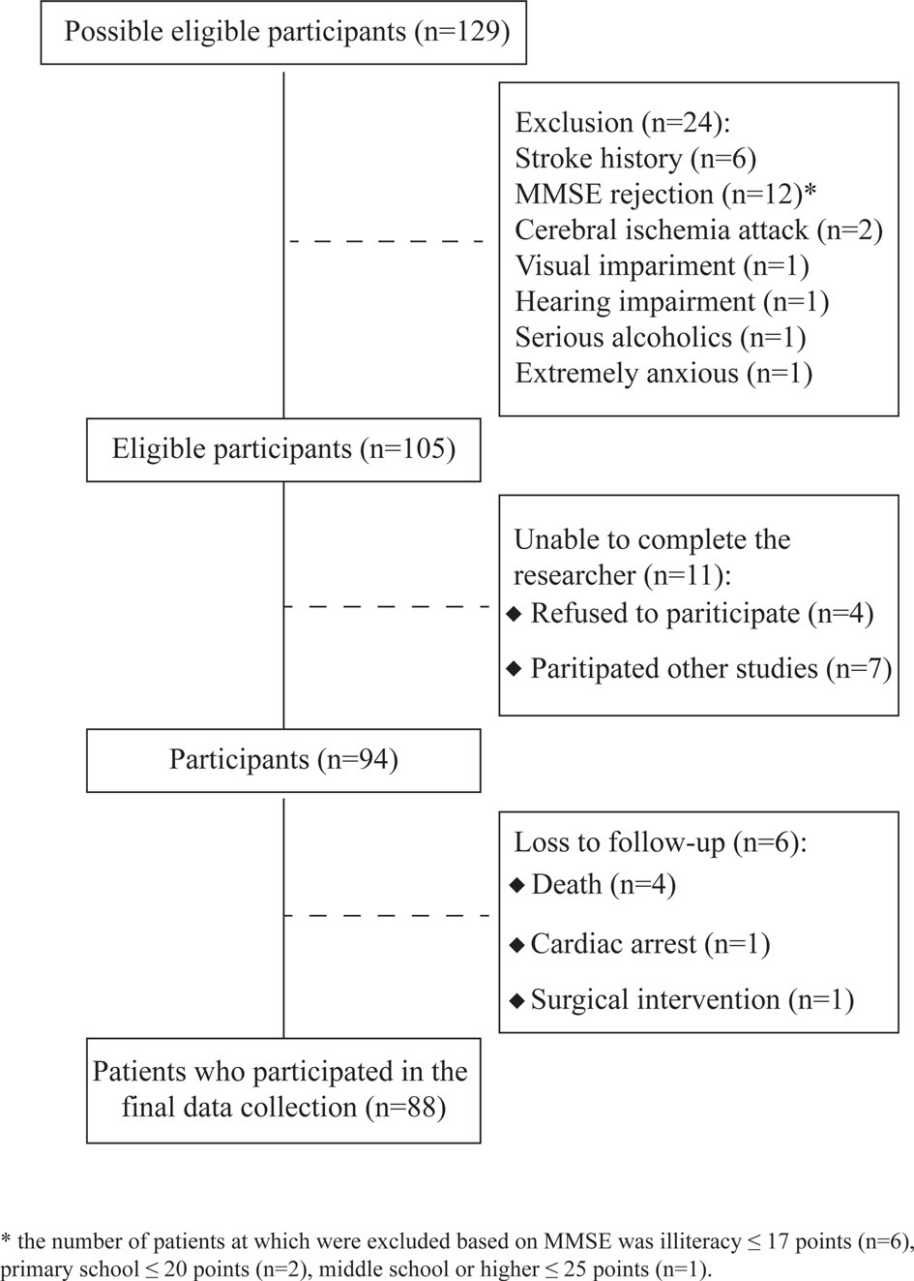
Schematic diagram of the case screening process

Patients were excluded for the following reasons: (1) preoperative cognitive impairment according to the test of MMSE (illiteracy ≤ 17 points, primary school ≤ 20 points, middle school or higher ≤ 25 points) [8]; (2) concomitant severe central nervous system diseases or history of mental illnesses (stroke, transient ischemic attack, severe anxiety disorder, drug addiction etc.); (3) history of brain surgery; (4) neuropsychiatric drugs therapy; (5) severe auditory or visual impairment causing the cognitive function tests cannot be performed; (6) other CVDs requiring surgery at the same time; (7) serious alcoholics; (8) refused to participate or already enrolled in other experimental studies. After the above exclusion, a total of 88 eligible subjects were screened out (Figure 1).

### Anesthesia methods

Oral administration of diazepam 0.1 mg/kg was given before the operation to maintain β-blockers or nitrates before surgery. All patients received 0.1 mg/kg of morphine and 0.3 mg of scopolamine 30 min before anesthesia. After entering the operating room, pulse oxygen saturation (SpO_2_), electrocardiogram (ECG), heart rate (HR) and non-invasive blood pressure (NBP) were monitored routinely. In addition, the peripheral venous access was opened and the invasive arterial pressure was monitored by a radial arterial puncture catheter under local anesthesia.

Anesthesia was induced by a dose of 0.1 mg/kg of midazolam, 0.2 mg/kg of etomidate, 0.5 μg/kg of sufentanil and 0.15 mg/kg of cisatracurium besilate through intravenous injection. Then the tracheal intubation performed mechanical ventilation. The respiratory frequency is 8–10 times/min, inhaled oxygen concentration (FiO_2_) is 60%, flow rate is 2 l/min, tidal volume 8–10 ml/kg, inhalation ratio 1:1.5 to 2, maintaining CO_2_ partial pressure at end of breath (P_ET_CO_2_) at 35–45 mmHg, bispectral index (BIS) depth of anesthesia 40–60. Target-controlled plasma infusion of propofol is 2.0–3.5 μg/ml and the sevoflurane for inhaled anesthesia with Minimal Alveolar Concentration (MAC) is 1.0. Static pump injection of remifentanil at a constant rate of 0.1–1 μg/(kg.min) to maintain anesthesia. Discontinuous injection of cisatracurium besilate, and added 0.5–1 μg/kg of sufentanil before incision and cardiopulmonary bypass (CPB). Automatic analgesia after surgery was performed by intravenous administration.

### Observation indicators

The patients were screened according to the level of cognitive impairment at 3, 7, 21, 90, 180 days with Spanish Telephone Interview for Cognitive Status (TICS-M). The occurrence of POCD in patients 21- and 180-days after surgery was determined by Neuropsychological Battery which were more intuitive, not influenced by culture, and took no longer than 30 min to complete. The battery included tests of verbal learning and memory function (Verbal Learning Test (VLT), immediate recall (VLT-A1), short delay recall (VLT-A2), long delay recall (VLT-A3) and long delay recognition (VLT-D)), response inhibition, selective attention or cognitive flexibility (Stroop Color Word Test (SCWT): SCWT-1, SCWT-2 and SCWT-3) [9], graphomotor speed and executive ability (Trail Making Test (TMT): TMT-A, TMT-B) [10], and cognitive processing speed and sustained attention (Symbol Digit Modalities Test (SDMT)). A brief detailed description of the above tests which included aim, method and scoring was listed as follows: the participants were given 16 common nouns and the total amount of number of words correctly repeated over the first three attempts was recorded as a score of VLT-A1+VLT-A2+VLT-A3. In detail, subjects in VLT-A1 were asked to recall as many words as possible in any order after the 16 common nouns is presented, and this procedure was repeated five times. After the above attempts, participants were presented with another 16 common words which were called ‘interference list,’ to measure proactive interference (PI). Half of the new list can be classified as same categories as the first 16 nouns; the other half of the items were totally different. After recall of interference list, participants were then asked to recall the first 16 nouns again (short delay recall, VLT-A2) to measure retroactive interference (RI) effects produced by the interference list. Twenty minutes later, participants were asked to recall the first 16 nouns after a retention interval (long delay recall, VLT-A3). After the other cognitive tests had been administered, the first 16 words were asked to recall under giving a clue (VLT-D) [11]. In the SCWT task, scoring was based on time (seconds) to finish three different trials: participants were expected to correctly identify the color of words written in black inks as soon as quickly (SCWT-1); naming the color words which does not mean any color (SCWT-2); point out the color of the ink in which a word means a different color was printed, e.g. the word ‘red’ is printed in blue ink (SCWT-3) [9]. The TMT A measures psychomotor speed and attention which involves participants connecting the randomly distributed numbers from 1 to 25 as soon as possible. TMT B provides a measure of mental flexibility (i.e. attention, switching ability and working memory) which requires the subject to connect 25 numbers and letters in an alternating pattern (1-A-2-B-3-C, etc.) [10]. In task of SDMT, page headed by a key that couples the single digits 1–9 with nine symbols was presented to participants. Score of written response and oral response task was sequentially determined by numbers of responses can be made in 90 s in which digit was correctly coupled with the above row symbol [12].

### χ^2^ test or *t* test were used to assess the following factors

1. Preoperative risk factors including age, sex, body mass index (BMI), smoking history, history of myocardial infarction, comorbidities (hypertension, diabetes, dyslipidemia, chronic renal insufficiency, thyroid dysfunction, aortic plaque, carotid artery stenosis and cerebrovascular disease).
2. Intraoperative risk factors including anesthesia time, length of surgery, surgical method and the rate of decline in intraoperative hemoglobin concentration (ΔHb). Three postoperative risk factors including tracheal catheter indwelling time, Visual Analog Scale (VAS), and the Systemic Inflammatory Response Syndrome (SIRS) score on postoperative 2 days.
3. Four SIRS criteria for scoring: (i) fever or hypothermia (temperature > 38 or < 36°C); (ii) tachycardia (heart rate > 90 min^−1^); (iii) tachypnea (respiratory rate > 20 min^−1^) or arterial CO_2_ partial pressure (PaCO_2_) < 32 mmHg; (iv) increased or decreased peripheral white blood cell count (>12 × 10^9^/l or <4 × 10^9^/l, or immature monocytes >10%). One point for each of the above four items, the total score was added up.

### POCD diagnostic criteria

TICS-M was only used to evaluate general postoperative cognitive function status of patients and was not included in the complete Neuropsychological Battery and POCD screen. A comprehensive set of cognitive function indicators were established for diagnosis of POCD: VLT-A (VLT-A1, VLT-A2 and VLT-A3 test scores), VLT-D, SCWT-1, SCWT-2, SCWT-3, TMT-A, TMT-B and SDMT. POCD was defined when at least two of the eight tests show a decreased score > 1.96 standard deviation (SD; of the whole group at baseline) [13,14].

### Statistical analysis

All data were expressed as frequencies, means ± SD or median ± quartile as appropriate. Student’s *t* test was applied to compare two groups of quantitative data which were normally distributed, and Mann–Whitney U test was performed to compare the non-normally distributed data. Frequencies were compared using chi-square test; Bonferroni method was applied to correct the *P*-value when comparing the multiple groups in pairs. All data were analyzed and processed by Windows software SPSS version 18.0 (IBM, U.S.A.), and value of *P*<0.05 was considered as statistically significant. The variables with *P*-value <0.05 (two-sided) on the univariate analysis were further tested by multivariate logistic stepwise regression analysis to adjust the confounding and determine the factors which were really associated with occurrence of POCD.

To determine the required sample size, the following criteria were taken into consideration: level = 0.05 (two-sided); statistical power = 0.8; proportion of cognitive dysfunction in control group = 35%; the estimated incidence of cognitive dysfunction in CABG group = 65% thus the required sample size was 102 (51 in each group). To accommodate an expected 10% dropout or lost to follow-up rate, the final enrollment was 112 patients [15,16].

## Results

### General information of patients

A total of 129 patients who had elective CABG during May 2015 to May 2017 in Qianfoshan Hospital were taken into consideration. Finally, 88 of them were selected as research subjects. Clinical statistical data can be found in Table 1.

**Table 1:** Population characteristics data of the study population (*n*=88)

Relevant factors	Ratio [*n* (%)]
Sex (male/female)	77/23
BMI	27.1 (3.7)
Active smoking	15 (17)
Myocardial infarction history	38 (43)
Hypertension	79 (90)
Diabetes	43 (49)
Hyperlipidemia	61 (69)
Chronic renal insufficiency	7 (8)
Hypothyroidism	6 (7)
Aortic plaque	16 (18)
Carotid artery stenosis	12 (14)
Cerebrovascular disease	17 (19)

### Diagnosis of POCD

TICS-M was used to evaluate the general cognitive function of patients, and the results showed that 69.8, 50.7, 47.9, 36.8 and 41.0% of patients had cognitive impairments at 3, 7, 21, 90 and 180 days after the surgery. At last, Neuropsychological Battery was used to diagnose POCD on 21 and 180 days.

### VLT

VLT scale test was used to investigate the verbal learning and memory processing ability of the subjects. The total score of the test (VLT-A1+VLT-A2+VLT-A3) showed that the incidence of POCD at 21 and 180 days after the surgery was 34.1 and 31.8%, respectively. The results indicated that the score was significantly higher in POCD patients than normal subjects (21 days after surgery: t = 4.646, *P*=0.001 < 0.01; 180 days after the surgery: t = 5.53, *P*=0.000 < 0.01). VLT-D test showed that the incidence of POCD at 21 or 180 days after the surgery was 38.6 and 34.1% respectively. VLT-D score of the POCD group are significantly lower than normal subjects (21 days after the surgery: t = 2.311, *P*=0.002 < 0.01; 180 days after the surgery: t = 2.846, *P*=0.001 < 0.01). All the data are shown in Table 2.

**Table 2:** Days 21 and 180 after the surgery VLT scale test, POCD incidence and scores 
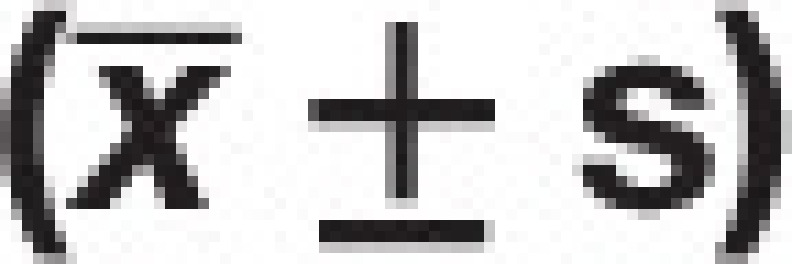

T	Group	VLT-A1+VLT-A2+VLT-A3		VLT-D	
		*n* (%)	VLT score	*n* (%)	VLT score
21 days	POCD (+)	30 (34.1)	32.1 ± 7.33^1^	34 (38.6)	8.57 ± 2.37^1^
	POCD (−)	58 (65.9)	43.23 ± 5.24	54 (61.4)	12.23 ± 4.59
180 days	POCD (+)	28 (31.8)	30.8 ± 6.94^1^	30 (34.1)	8.53 ± 3.68^1^
	POCD (−)	60 (68.2)	42.25 ± 6.73	58 (65.9)	13.21 ± 5.92

Abbreviations: VLT-A1, immediate recall of verbal learning test; VLT-A2, short delay recall of verbal learning test; VLT-A3, long delay recall of verbal learning test; VLT-D, long delay recognition of verbal learning test. ^1^*P*<0.01 POCD (+) *vs* POCD (−).

### SCWT

The SCWT test was used to assess the patient’s cognitive speed and inhibitory control capacity. The SCWT-1, SCWT-2 and SCWT-3 tests showed that the incidence of POCD was 14.8, 36.4 and 45.5% at 21 days after surgery. The incidence of POCD at 180 days after surgery was 12.5, 31.8 and 41.0%. The average time consumption of SCWT test in the POCD group was significantly higher than that of the normal group (21 days after surgery: t = 4.531, 8.271, 12.58, *P*=0.001, 0.000, 0.000 < 0.01; 180 days after surgery: t = 4.336, 7.92, 13.831, *P*=0.001, 0.000, 0.000 < 0.01). All the data can be found in Table 3.

**Table 3:** Days 21 and 180 after the surgery SCWT test, POCD incidence and scores 
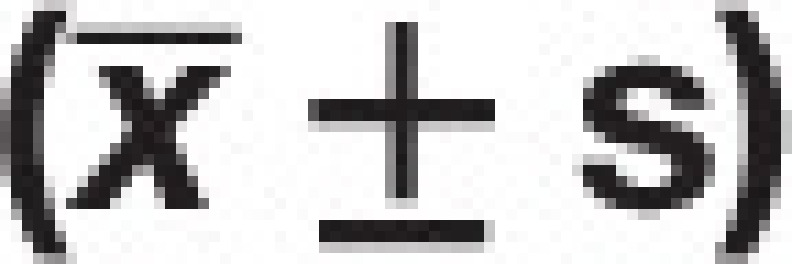

T	Group	SCWT-1		SCWT-2		SCWT-3	
		*n* (%)	Time/s	*n* (%)	Time/s	*n* (%)	Time/s
21 days	POCD (+)	13 (14.8)	19.54 ± 6.58^1^	32 (36.4)	33.28 ± 9.42^1^	40 (45.5)	69.42 ± 10.23^1^
	POCD (−)	75 (85.2)	10.27 ± 4.3	56 (63.6)	16.87 ± 6.28	48 (54.5)	33.28 ± 7.4
180 days	POCD (+)	11 (12.5)	18.23 ± 5.14^1^	28 (31.8)	36.51 ± 12.8^1^	36 (41.0)	72.3 ± 14.93^1^
	POCD (−)	77 (87.5)	9.9 ± 3.87	60 (68.2)	18.47 ± 6.84	52 (59.0)	30.15 ± 7.33

Abbreviations: T, time after the operation; SCWT-1, SCWT-2 and SCWT-3 indicated three groups of cards of different shapes. ^1^*P*<0.01 POCD (+) *vs* POCD (−).

### TMT

TMT scale test was used as an indicator of graphomotor speed and executive function. TMT-A and TMT-B test showed that the incidence of POCD at 21 days after the surgery was 13.6 and 15.9%, respectively; the incidence of POCD at 180 days after surgery was 11.4 and 10.2%, respectively. The average time spent in the TMT test in the POCD group was significantly higher than that in the normal group (21 days after the surgery: t = 4.331, 6.357, *P*=0.001, 0.000 < 0.01; 180 days after the surgery: t = 3.392, 7.839, *P*=0.001, 0.000 < 0.01). All the data can be found in Table 4.

**Table 4:** Days 21 and 180 after the surgery TMT test, POCD incidence and scores 
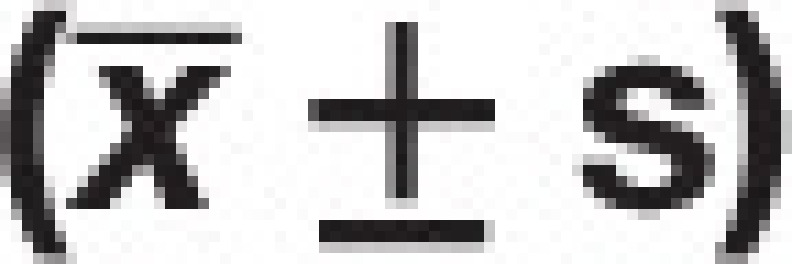

T	Group	TMT-A *n* (%)	Time/s	TMT-B *n* (%)	Time/s
21 days	POCD (+)	12 (13.6)	18.25 ± 5.33^1^	14 (15.9)	28.34 ± 6.52^1^
	POCD (−)	76 (86.4)	8.23 ± 1.14	84 (84.1)	12.52 ± 2.36
180 days	POCD (+)	10 (11.4)	17.37 ± 6.76^1^	9 (10.2)	27.25 ± 5.73^1^
	POCD (−)	78 (88.6)	8.96 ± 1.34	79 (89.8)	12.41 ± 3.07

Abbreviation: T, time after the operation. ^1^*P*<0.01 POCD (+) *vs* POCD (−).

### SDMT

SMDT test showed that the incidence of POCD at 21 days after the surgery was 22.7% and was 18.2% at 180 days. In SMDT writing test the correct number of POCD group was significantly higher than normal (21 days after the surgery: t = 3.24, *P*=0.001 < 0.01; 180 days after the surgery: t = 3.347, *P*=0.001, 0.000 < 0.01). In SMDT oral test, the number of correct oral expression in POCD group was significantly higher than normal group (21 days after surgery: t = 4.574, *P*=0.000 < 0.01; 180 days after the surgery: t = 3.874, *P*=0.001, 0.000 < 0.01). All the data can be found in Table 5.

**Table 5:** SMDT test, POCD incidence and number of correct symbol conversion at different times 
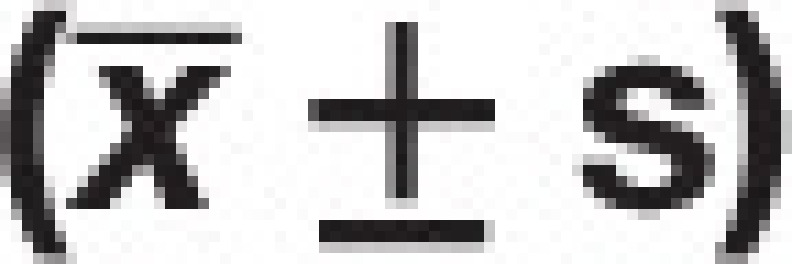

	*n* (%)	21 days after surgery		*n* (%)	180 days after surgery	
		NCW	NCO		NCW	NCO
POCD (+)	20 (22.7)	47.81 ± 10.11^1^	51.69 ± 12.08^1^	16 (18.2)	45.42 ± 8.85^1^	53.69 ± 10.92^1^
POCD (−)	68 (77.3)	62.44 ± 9.31	67.1 ± 8.67	72 (81.8)	61.28 ± 9.55	65.44 ± 6.9

Abbreviations: NCO, number of correct oral expression; NCW, number of correct writing. ^1^*P*<0.01 POCD (+) *vs* POCD (−).

### Incidence of POCD

According to the diagnostic criteria of POCD mentioned above in which at least two of the eight tests show a decreased score > 1.96 SD of the whole group at baseline. Twenty-three patients were diagnosed as POCD (26.1%) at 21 days after the surgery, among which, 74% of them were males and 95% of the patients were with the age ≥ 65. Furthermore, 20 patients were diagnosed as POCD (22.7%) 180 days after the surgery and 80% of them were males, and 90% of them are with the age ≥ 65.

In order to investigate the risk factors of the POCD at 21 days after surgery. Univariate χ^2^ analysis was performed firstly, and then Logistic multiple regression analysis was used for the subjects with *P*<0.05.

As to the preoperative variables, the results of univariate analysis revealed that age, aortic plaque, carotid artery stenosis and cerebrovascular disease have significantly effect on the occurrence of POCD in CABG patients (*P*<0.05) (Table 6). In addition, the anesthesia time and ΔHb which was a controlled factor during surgery were also related to the occurrence of POCD (*P*<0.05), however, the surgical methods and length of surgery have no significant effect on the occurrence of POCD after CABG (Table 7). At last, the analysis of postoperative variables showed that SIRS score may affect the occurrence of POCD in CABG patients (*P*<0.05), and the tracheal catheter indwelling time or VAS score has no significant difference between the two groups in which POCD happened or not (Table 8).

**Table 6:** The connection between postoperative cognitive functions and preoperative risk factors

Risk factors	Cognitive function		Total	Incidence (%)	χ^2^	*P*
	POCD(−)	POCD (+)				
						
Age						
<65	25	8	33	24.2		0.029^1^
≥65	33	22	55	40.0		
Total	58	30	88	34.1^1^		
Sex						
Male	51	17	68	25.0	0.20	0.655
Female	14	6	20	30.0		
Total	65	23	88	26.1^1^		
Smoking history						
<1 year	54	19	73	26.0	2.765	0.251
1 year ≤ age < 5 years	6	2	8	25.0		
≥5 years	5	2	7	28.6		
Total	65	23	88	26.1^1^		
Heart attack history						
Yes	29	9	38	23.7	0.208	0.648
No	36	14	50	28.0		
Total	65	23	88	26.1^1^		
Hypertension						
Yes	56	23	79	29.1		0.056
No	9	0	9	0.0		
Total	65	23	88	26.1^1^		
Diabetes						
Yes	30	13	43	30.2	0.731	0.393
No	35	10	45	22.2		
Total	65	23	88	26.1^1^		
Hyperlipidemia						
Yes	48	13	61	30.2	2.397	0.122
No	17	10	27	22.2		
Total	65	23	88	26.1^1^		
Chronic renal insufficiency						
Yes	5	2	7	28.6		0.591
No	60	21	81	25.9		
Total	65	23	88	26.1^1^		
Hypothyroidism						
Yes	5	1	6	16.7		0.503
No	60	22	82	26.8		
Total	65	23	88	26.1^1^		
Aortic plaque						
Yes	1	15	16	93.8		0.000^1^
No	64	8	72	11.1		
Total	65	23	88	26.1^1^		
Carotid artery stenosis						
Yes	2	10	12	83.3		0.000^1^
No	63	13	76	17.1		
Total	65	23	88	26.1^1^		
Cerebrovascular disease						
Yes	9	8	17	47.0		0.018^1^
No	56	15	71	21.1		
Total	65	23	88	26.1^1^		

^1^*P*<0.01 POCD (+) *vs* POCD (−).

**Table 7:** The connection between postoperative cognitive functions and intraoperative risk factors

Risk factors	Cognitive function		Total	Incidence (%)	χ^2^	*P*
	POCD (−)	POCD (+)				
Surgical methods						
Off-pump	27	9	36	25.0	0.041	0.840
On-pump	38	14	52	26.9		
Total	65	23	88	26.1^1^		
Length of surgery						
<3 h	4	0	4	0.0	3.826	0.148
3–4 h	57	19	76	25.0		
>4 h	4	4	8	50.0		
Total	65	23	88	26.1^1^		
<30%	29	4	33	12.1	14.687	0.001^1^
Anesthesia time						
30–40%	33	12	45	26.7		
>40%	3	7	10	70.0		
Total	65	23	88	26.1^1^		
<30%	29	4	33	12.1	13.330	0.001^1^
ΔHb (%)						
30–40%	33	12	45	26.7		
>40%	3	7	10	70.0		
Total	65	23	88	26.1^1^		

^1^*P*<0.01 POCD (+) *vs* POCD (–).

**Table 8:** The connection between postoperative cognitive functions and postoperative risk factors

Risk factors	Cognitive Function		Total	Incidence (%)	χ^2^	*P*
	POCD (−)	POCD (+)				
Tracheal catheter indwelling time						
<5 h	6	3	9	33.3	1.775	0.142
5–8 h	44	12	56	21.4		
>8 h	15	8	23	34.8		
Total	65	23	88	26.1^1^		
VLT score						
<1	24	8	32	25.0	0.059	0.971
1–3	32	12	44	27.3		
>3	9	3	12	25.0		
Total	65	23	88	26.1^1^		
SIRS score						
<1	39	7	46	15.2	9.517	0.009^1^
1–2	21	9	30	30.0		
>2	5	7	12	58.3		
Total	65	23	88	26.1^1^		

^1^*P*<0.01 POCD (+) *vs* POCD (–).

Considering our relatively small sample size and the subsequent low statistical power of multiple logistic analysis. We classified the statistically significant risk factors (*P*<0.05) mentioned above as dichotomous variable according to its distribution in each group (Table 9). Collectively, the risk factors including age, aortic plaque, carotid stenosis, cerebrovascular disease, anesthesia time, the rate of decline in intraoperative hemoglobin (ΔHb), SIRS scores and the cognitive function at 21 days after CABG were named as variable X1∼7 and Y respectively, and the assignment of different factors can be found in Table 10.

**Table 9:** Univariate analysis on measurement data of risk factors for POCD

Relevant factors	POCD group (*n*=23)	Non-POCD group (*n*=65)	*t*	*P*
Age	67.91 ± 6.42	58.3 ± 7.38	3.766	0.001^1^
Anesthesia time	4.23 ± 0.81	3.78 ± 0.66	3.03	0.006^1^
ΔHb (%)	38.45 ± 5.78	30.63 ± 7.83	3.484	0.002^1^
SIRS score	1.63 ± 0.79	0.83 ± 0.52	3.351	0.003^1^

^1^*P*<0.01 POCD (+) *vs* POCD (−).

**Table 10:** Possible risk factors and its assignments of POCD after CABG

Risk factors	Name	Assignment description
Age (year)	X1	<65 = 1, ≥65 = 2
Aortic plaque	X2	No = 0, Yes = 1
Carotid artery stenosis	X3	No = 0, Yes = 1
Cerebrovascular disease	X4	No = 0, Yes = 1
Anesthesia time	X5	<4 h = 1, ≥4 h = 2
ΔHb (%)	X6	<40% = 1, ≥40% = 2
SIRS score	X7	<2 point = 1, ≥2 points = 2
21 days of cognitive function	Y	Normal = 0, POCD = 1

To explore the independent risk factors for .POCD, multiple logistic regression analysis was applied to the above risk factors with a *P*-value <0.05. As shown in Table 11, all the population regression coefficients except for the constant term were not equal to 0, and the *P*-values were all less than 0.05, which suggested that the regression equation was valid. Among all risk factors, SIRS score (odds ratio (OR) = 1.284, 95% C.I. = 1.117–1.687), carotid artery stenosis (OR = 7.044, 95% C.I. = 1.317–37.687) and anesthesia time (OR = 1.043, 95% C.I. = 1.010–1.076) were the independent factors for POCD occurrence in 21 days after CABG (*P*=0.012, 0.023 and 0.010, respectively).

**Table 11:** Possible risk factors and their conections of POCD after CABG

		B	SE	Wald	df	Sig	Exp(B)	95% C.I. for EXP(B)
Step 1	X5	0.037	0.015	6.123	1	0.013	1.038	1.008–1.069
	Constant	−8.630	3.337	6.686	1	0.010	0.000	
Step 2	X7	0.237	0.231	1.056	1	0.037	1.267	1.159–1.382
	X5	0.046	0.016	8.076	1	0.004	1.048	1.015–1.082
	Constant	−8.591	3.375	6.478	1	0.011	0.000	
Step 3	X7	0.250	0.231	1.174	1	0.012	1.284	1.117–1.687
	X3	1.952	0.856	5.204	1	0.023	7.044	1.317–37.687
	X5	0.042	0.016	6.571	1	0.010	1.043	1.010–1.076
	Constant	−8.366	3.354	6.233	1	0.013	0.000	

B, partial regression coefficient; SE, standard error of partial regression coefficient Wald: value of χ^2^; df, degree of freedom; Sig, value of *P*; Exp (B), OR value.

## Discussion

The present study based on postoperative cognitive function assessment found that the incidence of POCD at 21- and 180-days after CABG was respectively 26.1 and 22.7%, and confirm that the risk of POCD increases with carotid artery stenosis, anesthesia time, and 2-day postoperative SIRS score. However, the surgical intervention which include on-pump or off-pump was no related to the risk of POCD, which is consistent with the findings of Van Dijk et al. [17]. Overall, our findings provide implications for the development of targeted interventions to prevent POCD after CABG surgery and shed light for the establishment of an effective behavior evaluation method of POCD. Furthermore, the incidence of POCD at 180 days after CABG suggested us that long-term POCD can occur after surgery of CABG, monitoring and therapeutic measures may need to be given as appropriate.

The relative higher rate of POCD in our study, 26.1% at 21 days and 22.7% after 180 days, may not reflect the true rate of POCD in general population undergoing surgery. The patients in our study are relative elders as they are prompt to suffer CVDs. In addition, continuing ill health and fraility, which might result in a reduced ability to modulate the neuroinflammatory and hypoxemia occurred during surgery [18]. Similarly, in our study the univariate analysis also showed that age factors had a certain impact on the occurrence of POCD in CABG patients which was consistent with the findings of previous studies [19].

It was reported that chronic cerebral hypoperfusion and increased blood pressure caused by carotid artery stenosis could result in the disruption of white matter integrity [20,21] and a decline in performance on cognitive tests. Silvestrini et al.’s study found that carotid atherosclerosis which accompanied with reduced cerebral blood flow could lead to brain energy metabolism dysfunction, reduce glucose utilization, loss of local acetylcholine receptors and finally result in local brain tissue ischemic injury and cognitive dysfunction [22]. Similarly, the result of Norkiene et al.’s study also showed that carotid stenosis could increase the incidence of POCD in CABG, which is consistent with the results of the present study [23].

It has been reported that general anesthesia may be one of the important factors that affect the postoperative cognitive function of patients, and it has a significant impact on postoperative memory, real-time calculation and logical cognitive ability, and can change the patient’s memory process. The prolonged anesthetic time leads to a corresponding increase in the amount of anesthetic drugs. The drug acts on the N-type cholinergic (nAch) receptors in the central nervous system and reduce the secretion of excitatory amino acids (EAA) in brain tissue, while EAA is associated with learning, memory and the occurrence and development of various acute and chronic brain injuries [24]. This study demonstrates that prolonged intraoperative anesthesia is one of the risk factors for POCD after CABG.

The study found that SIRS score on the second day after POCD group were higher than non-POCD group, suggesting that systemic inflammatory response is an important risk factor for POCD. The neuro-inflammatory response induces large number of inflammatory factors released. These inflammatory factors can directly affect the function of neurons and regulate neuronal intracellular pathways (such as brain-derived neurotrophic factor (BDNF)-mediated signaling pathways). This further affects brain and cognitive function. Hudetz et al. demonstrated that elevated inflammatory cytokines IL-6 and C-reactive protein (CRP) after CABG are important risk factors for POCD in the early and intraoperative periods [25].

Researches have shown that the use of on-pump surgeries leads to microembolism, abnormal cerebral oxygen metabolism and systemic inflammatory reactions and all these can damage the central nervous system and they are the main cause of brain injury after on-pump [26]. Off-pump can significantly reduce the incidence of brain stroke and fistula after CABG surgery, however large number of studies found that off-pump technology had no obvious advantage in reducing the incidence of POCD [27]. Van Dijk et al. found that the effects of on-pump and off-pump on postoperative cognitive function after surgery in the short-term and long-term were not significantly different [17], therefore, the relationship between CPB and POCD was not yet determined [28]. In this study, there was no significant difference in the incidence of POCD in the off-pump groups and on-pump groups. The main reason is that off-pump prevents CPB from damaging the brain but could not control other factors that may injure the brain. In addition, some unfavorable factors in CPB can be protective factors for the occurrence of POCD. For example, hypothermia in CPB may increase brain’s tolerance to hypoxia.

Many studies have shown that the metabolic diseases (such as hypertension, diabetes, dyslipidemia etc.) play an important role in cognitive function impairment. Hypertension can cause cerebral vascular remodeling and reduce cerebral neuromodulatory regulating function, leading to abnormal brain perfusion and lacunar infarction in perioperative patients, and it is an important factor in the occurrence of POCD [29]. Hudetz et al. found that patients with metabolic syndrome had a higher incidence of POCD than patients without metabolic syndrome [30]. In this study, univariate analysis found that there was a significant relationship between hypertension and the occurrence of POCD, but the multivariate analysis was negative. This may be related to other factors and preoperative control of blood pressure.

However, there are some limitations in the present study we would like to point out in order to improve future researches. Firstly, the present investigation has the inherent limitations of a retrospective, single-center cohort study, in which the recruited cases and clinical management can be the confounding variables and the analysis is confined to the existing data. Secondly, in order to obtain complete case data and meet the inclusion criteria, some cases were excluded, the sample size is relatively small. Hence, the low power of the test caused some of the variables were not included in the final multiple regression analysis. Furthermore, in the presented studies, the long-term cognitive function status of patients after CABG could not be studied. In consequence, the results are relatively biased and may affect the representativeness of the results.

## Conclusion

In conclusion, the incidence of POCD is relatively high at 21 days after CABG of approximately 26.1 and 22.7% still had POCD half-a-year after surgery, as such effective measures needed to strengthen monitoring and guide the therapeutic decision. Furthermore, patients with carotid artery stenosis, prolonged anesthesia time and systemic inflammatory response at postoperative 2 days are more likely to suffer from POCD, and early intervention in clinic is required in the above patients. Nevertheless, future research should focus on the specific determinants and underlying mechanisms for POCD.

## Abbreviations

CABG: coronary artery bypass grafting
CPB: cardiopulmonary bypass
CVD: cardiovascular disease
EAA: excitatory amino acid
MMSE: mini-mental state examination
OR: odds ratio
POCD: postoperative cognitive dysfunction
SCWT: Stroop Color Word Test
SDMT: Symbol Digit Modalities Test
SIRS: systemic inflammatory response syndrome
TICS-M: Telephone Interview for Cognitive Status
TMT: Trail Making Test
VAS: Visual Analog Scale
VLT: Vocabular/Verbal Learning Test
ΔHb: rate of decline in intraoperative hemoglobin concentration
